# Primary nasopharyngeal papillary adenocarcinoma: A CARE-compliant case report

**DOI:** 10.1097/MD.0000000000027729

**Published:** 2021-11-05

**Authors:** Chih-Hsuan Shen, Shih-Lun Chang, Sheng-Tsung Chang

**Affiliations:** a Department of Otorhinolaryngology, Chi Mei Medical Center, Tainan, Taiwan; b Department of Optometry, Chung Hwa University of Medical Technology, Taiwan; c Department of Pathology, Chi Mei Medical Center, Tainan, Taiwan.

**Keywords:** immunohistochemistry, nasopharyngeal papillary adenocarcinoma, nasopharynx neoplasm

## Abstract

**Rationale:**

Primary nasopharyngeal papillary adenocarcinoma is a rare nasopharyngeal neoplasm with a good prognosis and a low propensity for regional recurrence. To date, only few cases of primary nasopharyngeal papillary adenocarcinoma have been reported in the literature.

**Patient concerns:**

A 24-year-old female patient presented with intermittent hemoptysis and blood tinge nasal discharge.

**Diagnosis:**

An exophytic and pedunculated mass over the roof of the nasopharynx was found on nasopharyngoscope. Biopsy was done and the pathology confirmed well-differentiated primary nasopharyngeal papillary adenocarcinoma, strongly positive for CK7, and transcription termination factor 1; but negative for thyroglobulin. The final diagnosis was primary nasopharyngeal papillary adenocarcinoma, well-differentiated, pT1N0M0, stage I.

**Interventions:**

The patient underwent excision of nasopharyngeal tumor under sinuscopic assistance.

**Outcomes:**

: No local recurrence or distant metastasis was noted during the 6 months of follow-up.

**Lessons:**

We aim at highlighting the importance of a thorough differential diagnosis of nasopharyngeal tumor. Further investigation is still needed for providing evidence to standardize the treatment protocol.

## 1 Introduction

Primary nasopharyngeal papillary adenocarcinoma is an extremely rare pathological type of nasopharyngeal carcinoma (NPC), accounting for less than 0.48% of all malignant nasopharyngeal neoplasms.^[1,2]^ It originates from the epithelium of the nasopharynx and most commonly occurs in the posterior or roof of nasopharyngeal walls. Histologically, primary papillary adenocarcinoma may mimic papillary thyroid carcinoma (PTC) for which immunohistochemistry (IHC) stain plays a vital role in supporting the diagnosis.^[3]^ Histological grade of the tumor is also important for treatment strategy and currently, there is no standard protocol for well-differentiated primary papillary adenocarcinoma. However, excellent prognosis and rare recurrence were noted with an appropriate surgical management, including transnasal endoscopic surgery.^[2–5]^ Herein, we present a rare case of a 24-year-old female with primary nasopharyngeal papillary adenocarcinoma.

## 2 Case report

A 24-year-old female patient was referred to our otolaryngology outpatient department with complaints of intermittent hemoptysis and blood tinge nasal discharge for 2 years. She had neither epistaxis, body weight loss, dyspnea, dysphagia, nor foreign body sensation over throat. She denied any history of facial trauma, past medical or surgical history. She had no exposure to cigarettes, alcohol, or betel nuts. The patient also denied any relevant family history.

**Figure 1:**
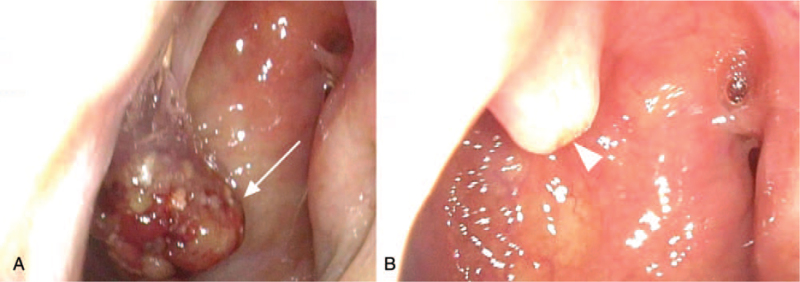
Nasopharyngoscopy. An exophytic, pedunculated tumor, sized 1.4 cm × 1.0 cm × 0.8 cm, at the roof of the nasopharynx. (A) prebiopsy (arrow). (B) postbiopsy (arrowhead).

**Figure 2:**
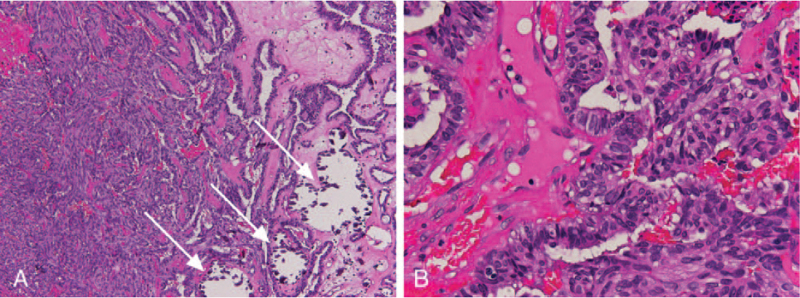
The tumor displayed papillary structure with fibrovascular cores lined with cuboidal or columnar epithelium; eosinophilic cytoplasm and epithelial cells with round to oval nuclei; and psammoma bodies (arrows) (A, Hematoxylin and eosin, 40×. B, Hematoxylin and eosin, 200×).

**Figure 3:**
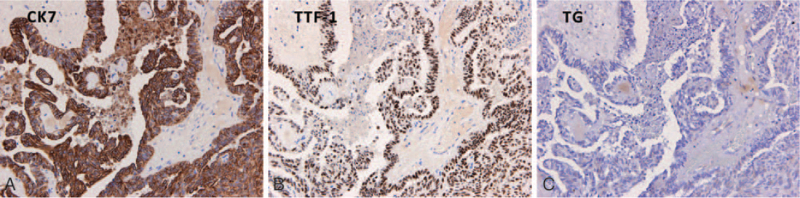
Immunohistochemical study showed the tumor cells are strongly positive for CK7 and TTF-1 (A and B), and negative for TG (C) (IHC stain, 200×). TTF-1 = transcription termination factor 1, TG = thyroglobulin.

Physical examination revealed no palpable cervical lymph nodes or other abnormalities. During the first nasopharyngoscopy, a bulging mass was found at the roof of nasopharynx. The pedunculated mass, sized 1.4 cm × 1.0 cm × 0.8 cm, was exophytic with a smooth nasopharynx mucosal surface (Fig. 1). Nasopharynx biopsy was then performed and the pathology report confirmed the diagnosis of well-differentiated nasopharynx papillary adenocarcinoma (Fig. 2). The tumor cells exhibit mild to moderate pleomorphism with round to oval nuclei and psammoma bodies can be identified focally. Immunohistochemically, the tumor cells express CK7 and transcription termination factor 1 (TTF-1) but not thyroglobulin, which supports the diagnosis (Fig. 3). Laboratory findings were within normal limits, including Epstein–Barr virus viral-capsid antigen IgA.

**Figure 4:**
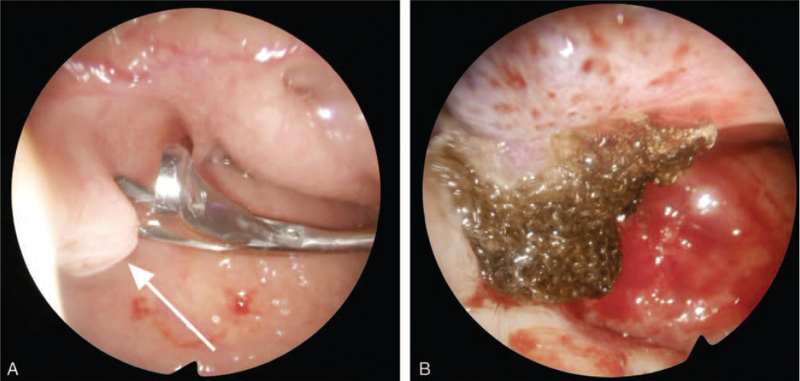
(A) Sinuscopy during operation of excision of nasopharyngeal tumor (arrow). (B) The tumor was completely excised with oxidized regenerated cellulose (Surgicel) packing.

Due to suspicious metastatic origin, a series of examinations for tumor staging was arranged, including chest X-ray, chest computed tomography, nasopharynx magnetic resonance imaging (MRI), abdominal sonography, esophagogastroduodenoscopy, whole body bone scan and positron emission tomography. The nasopharynx MRI revealed no definite abnormal enhancing mass lesion at the nasopharynx and absence of neck nodal metastasis, stage TxN0. Chest computed tomography reported inflammatory nodules in the right upper and lower lung, for which metastasis is less likely. The results of the positron emission tomography showed neither significant increased fluorodeoxyglucose avidity in the nasopharynx nor regional or distant metastasis. Excision of the nasopharyngeal tumor under sinuscopic assistance was then performed. The remnant stalk of the tumor was smoothly excised and no residual adenocarcinoma was found from the pathology report (Fig. 4).

The final diagnosis was primary papillary adenocarcinoma of nasopharynx, well-differentiated, pT1N0M0, stage I, with the follow-up period being 6 months to date. There is no local tumor recurrence noted in the nasopharynx under endoscopy. Nasopharynx MRI which was done 3 months after operation also revealed no recurrence.

## 3 Discussion

Primary nasopharyngeal papillary adenocarcinoma is rare, accounting for less than 0.48% of all malignant nasopharyngeal neoplasms.^[1,2]^ The most common malignancy in the nasopharynx is NPC, which histologically reveals nonkeratinizing and keratinizing squamous cell carcinomas without glandular differentiation. Another type of NPC is primary salivary gland-type, which involves 2 subtypes: adenoid cystic carcinoma and mucoepidermoid carcinoma.^[3,6]^

Primary nasopharyngeal papillary adenocarcinoma originates from the epithelium of the nasopharynx. To the best of our knowledge, only a few cases of primary nasopharyngeal papillary adenocarcinoma have been reported in the literature. Patients range in age from 7 to 77 years, and the incidence rate is higher in female, with a female to male ratio of 1.5:1. The tumors range from 0.3 cm to 4.0 cm and occur most commonly in the posterior or the roof of the nasopharyngeal walls. As for the most common symptoms, blood tinge rhinorrhea or even epistaxis is reported.^[3,5,7]^ In the present case, demographic characteristics, including age, gender, chief complaint, tumor size, and location, match the previous reports.

Macroscopically, the tumors are usually exophytic with a papillary, nodular, or polypoid appearance. They can be soft or granular.^[8]^ This special type of nasopharyngeal adenocarcinoma was first published from Wenig et al^[9]^ and named as a thyroid-like low-grade nasopharyngeal papillary adenocarcinoma. Owing to its low-grade morphology and indolent clinical behavior that differ from common adenocarcinomas, the 2005 World Health Organization classification system classified nasopharyngeal papillary adenocarcinoma as malignant epithelial tumors of the nasopharynx.^[10]^

Microscopically, according to previous literature, the tumor cells may have the following features: papillary structure with fibrovascular cores lined with cuboidal or columnar epithelium; eosinophilic cytoplasm and epithelial cells with round to oval nuclei; tiny nucleoli and a vesicular or relatively clear chromatin pattern; spindle tumor cell component; and psammoma bodies.^[5]^ Since the histopathology of primary nasopharyngeal papillary adenocarcinoma is similar to PTC, an IHC stain is critical in providing an accurate diagnosis.^[3]^ Positive cytokeratin staining revealed that the tumors arose from nasopharynx mucosal epithelium rather than from submucosal glands. TTF-1 is aimed to be specific to the thyroid gland and the lung. As primary nasopharyngeal papillary adenocarcinoma usually shows positive immunohistochemical reactivity for TTF-1, negative staining of thyroglobulin can offer evidence to distinguish between primary nasopharyngeal papillary adenocarcinoma and PTC. Negativity for CK20, CD15, S-100, P40, P63, CK5/6, CDX-2, prostate-specific antigen, and positivity for CK7, CK19, vimentin, and EMA on IHC can also support the diagnosis.^[1,2,5]^ In our case, the microscopic and immunohistochemical findings were consistent with these features.

The pathogenesis of the primary nasopharyngeal papillary adenocarcinoma remains controversial. Most nasopharyngeal neoplasms originating from the nasopharyngeal epithelium are often associated with the Epstein–Barr virus. However, based on previous literature, there is no known association between primary nasopharyngeal papillary adenocarcinoma and the Epstein–Barr virus.^[1,2,7,11]^ As for gene mutants, KRAS, NRAS, BRAF, EGFR, and ALK have reported as negative in previous studies.^[5,6,12]^ Due to the limited number of cases and studies, further investigation is needed to clarify the pathogenesis.

For the treatment, due to the rarity of nasopharynx papillary adenocarcinoma, there is still no standard treatment protocol. Whether surgical treatment alone, solely radiotherapy or chemotherapy, or surgery combine with radiotherapy or chemotherapy is still controversial. Clinical stage and histological grade of the tumor also play important roles in decision of treatment strategy.^[2,5]^ According to the literature review, surgical excision, including endonasal endoscopic excision, is the most appropriate treatment for well-differentiated primary nasopharyngeal papillary adenocarcinoma. Most patients have good prognosis with no recurrence and even revised endoscopic surgery showed successful outcome for recurrent disease.^[2–5]^ While the role of radiotherapy and chemotherapy have been mentioned in several studies, adjuvant radiotherapy is suggested for patients with incompletely resected tumors.^[2]^ Several studies also reported photodynamic therapy as a new treatment option but controversy exists due to small sample size and limited number of studies.^[2]^ Moreover, well-differentiated adenocarcinoma usually show poor response to radiotherapy or chemotherapy, revised surgery should be considered for patients with residual tumors or recurrence.^[2,5]^ In our young patient, pathologic report revealed well-differentiated nasopharynx papillary adenocarcinoma with no residual tumor cells postoperatively, thus, no adjuvant radiation therapy or chemotherapy was prescribed. There is no local recurrence or distant metastasis during a 6 months follow-up period.

## 4 Conclusion

Primary nasopharyngeal papillary adenocarcinoma, is a rare and low-grade neoplasm with a favorable prognosis and a low propensity for local recurrence and distant metastasis. Pathologists and clinicians should be aware of distinguishing this types of neoplasm accurately due to its similar morphology to PTC. Surgical excision, including endonasal endoscopic excision was an effective treatment with good outcome, especially for patients with well-differentiated primary nasopharyngeal papillary adenocarcinoma. Further investigation is still needed to provide evidence for a standardized treatment protocol.

## Author contributions

**Resources:** Shih-Lun Chang, Sheng-Tsung Chang.

**Supervision:** Shih-Lun Chang.

**Writing – original draft:** Chih-Hsuan Shen.

**Writing – review & editing:** Chih-Hsuan Shen, Shih-Lun Chang.
